# Critical Appraisal and Masculine Authority: The Boys Clubs’ Derogatory Method of Reading Canadian Feminist Speculative Fiction

**DOI:** 10.1177/17499755211062654

**Published:** 2022-07-05

**Authors:** Marie-Lise Drapeau-Bisson

**Affiliations:** University of Toronto, Canada

**Keywords:** boys’ club, Canada, cultural intermediaries, feminism, feminist literature, gender, hegemonic masculinity, method of reading, reception

## Abstract

Culture scholars have shown that cultural intermediaries play a crucial role in the reproduction of inequalities in consecration (Corse and Westervelt, 2002; Maguire Smith and Matthews, 2012; Miller, 2014; Ridgeway, 2011; Steinberg, 1990 cited in Bourdieu, 2010). However, the analysis of gender inequalities in reception and canonization has focused on individual bias, neglecting the contribution of scholars of hegemonic masculinity about the importance of patterned practices in the reproduction of men’s dominance over women (Connell and Messerschmidt, 2005). Given that art worlds are not settings where typical markers of hegemonic masculinity are valued, such as money and physical prowess, what are the tools of hegemonic masculinity in art worlds? I answer this question through a comparative analysis of the reception of two iconic Canadian feminist novels: *L’Euguélionne* (2012 [1976]) by Louky Bersianik and *The Handmaid’s Tale* (1985) by Margaret Atwood. Building on feminist scholarship, I find that the discursive apparatus of hegemonic masculinity in art worlds consists of a derogatory method of reading employed by critics in newspapers. This method of reading is founded on three discursive components: (i) a reductive reading of feminist politics; (ii) a man-centred assessment of feminism and (iii) a questioning of women’s creative credibility which belittles the contribution of feminist authors. By translating the concept of boys’ club (Delvaux, 2019) and identifying its derogatory method of reading, I propose a framework that illuminates how critical appraisal shapes discursive resources available for both professional and non-professional readers to draw upon for evaluation and classification of women’s cultural productions and feminist engagements.

## Introduction

In a recent study on women in the Québec literary field, Boisclair (2019) exposes how women are granted less visibility than their male counterparts, and how their work is discussed differently in critics’ appraisal. She finds that women’s literary production gets overwhelmingly described as ‘delicate’ and ‘sensitive’, compared to a lexicon of creative genius for men, with descriptors like ‘powerful’, ‘masterful’ and ‘remarkable’ (Boisclair, 2019: 25) Similarly, art-world professionals from other national contexts have lamented the hegemonic character of art criticism. Notably, Méndez Berry and Yang, in their 2019 opinion piece in the *New York Times*, criticized the lack of diversity amongst art critics reviewing the Whitney Biennial survey of American art, arguing that the predominantly white and male critics lacked the necessary racial literacy to interpret the works of the diverse artistic representation of the exhibition.^1^ Therefore, while art worlds may be diversifying, some aspects of their discourses do not seem to be transforming.

Cultural sociologists have well documented the crucial role cultural intermediaries (CIs) play in the reproduction of inequalities, be they based on class, gender or race (Bourdieu, 2010; Corse and Westervelt, 2002; Maguire Smith and Matthews, 2012). As arbiters of taste in the reception, interpretation and evaluation of artistic production across art worlds, CIs’ judgement is decisive – they can make or break a career, create or destroy a *chef d’oeuvre*. In terms of gender inequalities, research has shown that what is coded as feminine, be it artistic work or literary genre, is devalued (Atkinson, 2016; Lieberson et al., 2000; Miller, 2014; Mullin, 2003; Peterson, 2003). This line of research implies that gender disparities in consecration and canonization are the product of the individual bias of critics which somewhat changes over time, depending on context (Corse and Westervelt, 2002). However, scholars of hegemonic masculinity have demonstrated the importance of patterned organizational practices that rely on traditional definitions of masculine power in the reproduction of men’s dominance over women (Connell and Messerschmidt, 2005). Given that art worlds are not settings where typical markers of hegemonic masculinity are valued, such as money and physical prowess, what are the tools of hegemonic masculinity in art worlds?

Beyond the ascendency of some men over women in daily relations, the lens of hegemonic masculinity invites us to analyse how elites preserve their power and exclusivity through normalized gendered practices (Delvaux, 2019: 25). Since the legitimacy of elites in art worlds is articulated through critical appraisal, I ask: How is hegemonic masculinity articulated in art worlds’ reception? How do its discursive practices transform over time? To answer this question, I examine the evaluative discourse of literary criticism in newspapers. I compare the reception of two Canadian feminist novels that share a common trajectory to fame: Louky Bersianik’s *L’Euguélionne*^2^ (1976) and Margaret Atwood’s *The Handmaid’s Tale*^3^ (1985). While both novels were commercial successes and have become iconic, I find several critiques that cast doubt over their literary value. To analyse this phenomenon of joint consecration and disparagement, I bridge gender and reception scholarly works. I draw on the concept of the boys’ club, a feminist analytical tool developed by Québec scholar Delvaux (2019), to characterize the gendered character of the social orchestration and cultural matching revealed by the literature on critical appraisal and CIs. Building on DeVault’s (1990) work on gendered methods of reading in reception, I find that the boys’ club of literary criticism uses a derogatory method of reading that belittles the contribution of feminist authors. This method of reading consists of three discursive components: (i) a reductive reading of feminist politics; (ii) a man-centred assessment of books’ feminism and (iii) a challenge to women’s creative credibility.

Based on these findings, I argue that the derogatory method of reading consists of coercive gendering, a type of mechanism of invisibility (Connell and Messerschmidt, 2005: 834) that removes gender as a core criterion in critics’ evaluations. This means that gender discrimination in literary criticism persists even as gender is not explicitly discussed in evaluation. Published after important waves of feminist mobilization,^4^ discriminatory gendering in the two books’ reception, that is, discrimination which is ‘visible and measurable and hence legally sanctionable’ (Alacovska, 2017: 392) is no longer socially acceptable. Such coercive gendering helps us to understand not only gender exclusions, but also the process through which some women and versions of feminism are worthy of literary consecration, while others are interpreted as unpalatable.

Social justice movements confront hegemonic ideas associated with gender, race and sexuality, and it is common for such confrontations to be explored through art (Roy, 2010). While published 10 years apart, both novels encapsulate important moments for second-wave feminism in Canada’s two linguistic contexts – a flourishing period of feminist artistic productions for *L’Euguélionne* and a pivotal moment for reproductive justice for *The Handmaid’s Tale*. Yet, the decades that followed the publication of both books are known as hostile to feminist mobilization, with challenges ranging from government cuts for women’s programmes and to anti-choice counter-movements (Rodgers and Knight, 2011), to violence against women (van Wormer, 2008). The analysis of these two books’ critical appraisal thus speaks more broadly to the ways in which art worlds themselves cope with challenges and social transformations carried out by movements. In the case of the two iconic Canadian feminist books, critics are slow to adapt. *L’Euguélionne* and *The Handmaid’s Tale* are celebrated works and yet, as the findings will reveal, they are belittled by critics who interpret the authors as summarizers of feminist politics rather than theorists, and as unreliable witnesses of the speculative futures they create. I posit that because of CIs’ pivotal position in reception, their derogatory method of reading has implications for collective repertoires available to interpret women’s artistic work and political engagements. This article thus proposes a framework for the study of other characteristics of hegemonic boys’ clubs like whiteness, heteronormativity and Eurocentrism and how they shape cultural politics through reception.

## Gendered Readings and the *Boys’ Club* of Literary Criticism

I review the literature on critical appraisal through the lens of DeVault’s method of reading. This concept draws our attention to the interplay between personal, historical and organizational factors that shape the gendered discursive work of critics. I first explain in more detail the concept of method of reading and what it implies for our understanding of journalistic criticism, and then use it to review the critical appraisal literature along three lines: individual reading lens, historical context and professional objectives. Doing so draws a portrait of journalistic criticism as boys’ club, a term I borrow from feminist scholar Martine Delvaux; a group, traditionally dominated by men, orchestrated around gendered notions of credibility and whose status grants them power over who belongs, and who does not.

### Method of Reading: Reception, Standpoint, and Representation

The concept of method of reading by Marjorie DeVault (1990) bridges standpoint theory and Becker’s approach to reception as involving selection, translation and arrangement in users’ accounts of cultural products (1990: 905). DeVault proposes this concept in her analysis of the reception of Nadine Gordimer’s novel, *The Late Bourgeois World* to capture how audiences’ gendered experiences, historical context and professional objectives shape their accounts of the novel; what they decide to emphasize in their reading and evaluation of the novel, in particular their interest (or lack thereof) in the main female character’s life and story. DeVault differentiates between three methods of reading of Gordimer’s novel. First, male reviewers from the mid-1960s read the novel in terms of South African politics and rejected the female protagonist’s story as meaningful as they lacked the necessary background knowledge and experience to find interest in Liz’s story, ‘an intelligent and introspective single woman’ (DeVault, 1990: 917). They instead found meaning in the male characters’ stories and attributed the lack of meaning of the female character to flawed writing by the author, thus evaluating the novel negatively (DeVault, 1990: 917). In contrast, male readers of the late 1970s and early 1980s, reading after anti-racist and feminist mobilizations of the 1960s accepted Liz as the main character, but gave little importance to the details and concerns of her story. Feminist scholarly readers of the same era produced a third reading that identified Liz’s experience as ‘a point of interest in her own right’ (DeVault, 1990: 903). These different interpretations of the same book also involved different evaluations, used in turn to make an assertion about racial or feminist politics.

The concept of method of reading captures the impact of CIs’ reception work. Indeed, DeVault conceptualizes a method of reading as an interpretive activity, which means ‘a concern with the joint activities involved in producing representations and the activities that develop from using representations’ (DeVault, 1990: 889). DeVault’s concept propels us to take seriously what readers do with texts and the ethical impact of their readings, beyond the accumulation of capital (Thumala Olave, 2018). This approach situates critics as important actors in reception, while also considering their involvement in the production of cultural representations. Unlike other bridging approaches of cultural reception and production which attend to the ways in which dynamics of reception resemble the field of production (Chong, 2020) or how objects travel through these fields (Childress, 2017),^5^ I embrace reception studies’ interest in meaning-making through reading and look at how the accounts of the books themselves constitute representations of society that make arguments, much like maps, graphs and plays (Becker, 2007). The three components of the method of reading – personal lens, historical setting and organizational context, each reviewed later in this article – are thus used not only to understand to whom or what CIs confer (or withhold) literary legitimacy, but also to capture how gender shapes CIs’ making of representations though reception.

### Gender and Individual Bias

Cultural sociologists have well documented the effect gendered biases have on women artists, ranging from underrepresentation and lower pay (Miller, 2016), to differentiated treatment when they age and even after death (Hearsum, 2016; Lang and Lang, 1993). In terms of critical appraisal, this translates to gender-based symbolic devaluations of many kinds: ‘Women artists, musicians, and writers describe being taken less seriously than men, being treated as novices when they are actually skilled professionals, and having to work harder than men for equal recognition’ (Cowen, 1996; Leonard, 2007; Midler, 1980; Miller, 2014 all cited in Miller, 2016: 124). These devaluations concern the issue of creative credibility, as exposed by Solnit’s lauded piece *Men Explain Things to Me* (2014).

Solnit (2014) tells the story of a man she meets at a party, amusingly named Mr Important, who uses a *New York Times* review to explain to her ‘this important book’ which happens to be one she has authored. His explanation persists even as Solnit’s friend intervenes with explanations of Solnit’s authorship, which Mr Important cannot hear. Solnit’s essay highlights Mr Important’s imperviousness; his confidence about a book he had not read trumped Solnit’s expertise about a book she had written. As Solnit writes, such anecdotes make obvious forces that are hard to identify, like ‘an anaconda that’s eaten a cow or an elephant turd on the carpet’ (Solnit, 2014: 4). The forces underscored here are those that challenge women’s credibility, telling them ‘that they are not reliable witnesses to their own lives, that the truth is not their property, now or ever’ (Solnit, 2014: 7). Solnit’s essay reveals how gender influences whose reality we find credible and valid. In art worlds, where symbolic value is the main currency and legitimacy is a pre-requisite for consecration, the gendered character of credibility is a significant impediment to the success of some women artists.

### Gender and Historical Context

Reviewers’ gendered readings are also a product of their historical setting since the context, and the ‘intellectual resources’ it affords reviewers (Corse and Westervelt, 2002), shape the interpretation and evaluation of texts. As DeVault explains, ‘the early reviewers [of *The Late Bourgeois World*] wrote in the mid-1960s, before the women’s movement became a political force, and their writing reflects the unconscious but pervasive sexism of the time’ (1990: 897). Similarly, Corse and Westerwelt show that interpretive strategies used to evaluate the novel *The Awakening* by Kate Chopin changed through time. At the turn of the century (1899), it was first evaluated as immoral, then between 1950 and 1979 as a quest for self-identity and autonomy, and finally, since 1980, as belonging in the American literary cannon.

Beyond providing intellectual resources, the context also changes the dispositions of the readers. For *The Late Bourgeois World*, some critics writing in a changed South African context where anti-racism was an inevitable social force came to the book with political concerns. For instance, a scholarly reviewer developed an anti-racist analysis and focused on the issue of white involvement, an increasingly important issue in mid-1960s South African politics (DeVault, 1990: 901–902 ). Another example consists of a feminist reading, developed by a feminist scholar in a specialized literary journal, that focused on the integration of public and private life of Liz. She thus not only took Liz’s story seriously, but also uses her story to explore a contemporary feminist issue (DeVault, 1990: 902). This demonstrates how reviewers attend to aspects of the novel that seem relevant for the political context in which they read.

### Gender and Organisational Practices

Some organizational features distinguish the work of critics from that of scholarly reviews presented in the previous paragraph. As cultural intermediaries, critics’ judgments are ‘particularly consequential because they have the formal power to confer visibility, position, or material reward’ (Merriman, 2017: 441). As ‘culturally sanctioned connoisseurs’ (Chong, 2011: 64), their work is imbued with a moralistic tone as they determine between the artists and art works deserving and undeserving of attention and consecration (Becker, 2008). Journalistic critics are particularly interested in summary and evaluation, in contrast with scholarly critics whose articles focus on interpretation of literary texts, often in relation to the literary canon (DeVault, 1990). Published in newspapers and magazines, journalistic criticism typically reviews new or contemporary fiction. While insufficient for consecration, Chong (2011: 66) reports that getting the attention of those newspaper and literary critics is a predictor of a second book contract, selection for scholarly analysis and a ‘step on the road to being consecrated as a high-culture novelist’.

Critics, like other cultural intermediaries, work in a context ridden with uncertainty about assessment criteria (Childress and Nault, 2019), what Chong (2020) terms epistemological uncertainty. To palliate for the absence of objective standards in critical appraisal, critics often ‘attune their literary judgments to approximate the opinions of their peers’, a process called social orchestration (Chong, 2011: 66). This leads to a reliance on other tools in the evaluation of creative products, such as racial and ethnic categories. The over-reliance on personal experience is problematic for women as it means a greater reliance ‘on intuitive, unexamined criteria such as gender stereotypes’ (Miller, 2014; Ridgeway, 2011; Steinberg, 1990 cited in Miller, 2016: 124). This matters for things like grant allocations, slots at festivals, as well as media attention (Miller, 2016: 124–125). Indeed, Chong has shown how ‘fewer reviewers [are] willing to give attention to books by women’ because they fear hurting authors already facing obstacles on the basis of gender (2020: 138). The epistemological uncertainty of reviewing thus creates a greater reliance on individual characteristics, such as gender and race, and personalized approaches to structural problems of representation in reviewing.

CIs’ reliance on personal experience as an ‘institutionally entrenched and legiti-mate decision heuristic’ (Childress and Nault, 2019: 121) is problematic when the demographics of CIs significantly differs from that of the authors and artists of the cultural products they publish, edit and review. Building on Rivera’s article on cultural matching in hiring that found gender and race to be ‘powerful sources of interpersonal attraction and evaluation’ (Rivera, 2012: 1002), Childress and Nault (2019) show that publishers choose to ‘pass’ when there is no ‘fit’ between their white and/or masculine experience of the world and the characters the books showcase. These individual ‘passes’ demonstrate critics’ personalized problem-solving identified by Chong (2020) which leads to a disconnect between the structural power granted to critics and the uncertainty-driven, individual responses to questions such as diversity in the literary field (2020: 137). The work of critics is thus characterized by social orchestration where critics, predominantly white and male, use their personal experiences as a yardstick for selection and evaluation of cultural products.

To characterize professions traditionally exercised by white men and where membership, talent and legitimacy is defined along gendered lines, Québec feminist scholar Martine Delvaux proposes the concept of ‘boys’ club’. Taking roots in the British tradition of the Victorian private clubs, a *boys’ club* is: ‘an organization that traditionally excludes women and is controlled by men’ (Delvaux, 2019: 25, my translation). When applied to a variety of fields, ranging from media production to architecture, to city planning and politics, the boys’ club as an analytical device helps scholars identify masculine orchestration in fields where it is rendered neutral. While boys’ clubs no longer assert their economic and political affluence by meeting in a physical setting, like a country club or a cigar lounge, its members remain nonetheless ‘connected together – dependent upon one another, relating to each other, and unified by a common interest, a shared belief, an ideal to which everyone adheres’ (Delvaux, 2019: 25). In fact, recalling Solnit’s example about the masculine character of creative credibility, we might talk of the boys’ club of literary critics as united around a common *dis*belief – a disbelief that women can be authors, even of their own books.

The concept of the *boys’ club* encapsulates the organized character of gender regimes at the heart of the hegemonic masculinity scholarship. Hegemony does not imply total control or dominance, but rather ongoing subordination of certain non-dominant groups through ascendency embedded in institutions and cultural processes (Connell, 1987: 184). To be sustained, hegemonic masculinity must come with practices that ‘embody a successful collective strategy in relation to women’ (Connell, 1987: 186). This strategy involves, as in Gramsci’s original concept of hegemony, actors who ‘regulate and manage gender regimes; articulate experiences, fantasies and perspectives, reflect on and interpret gender relations’ (Connell, 1983 in Donaldson, 1993: 646). I posit that cultural intermediaries are such actors who regulate gender regimes in art worlds – they are, in Gramsci’s terms, the organizing intellectuals of art worlds.

Scholars of masculinity have highlighted how hegemonic masculinity is not fixed ‘always and everywhere the same. It is, rather, the masculinity that occupies the hegemonic position in a given pattern of gender relations, a position always contestable’ (Connell, 1995: 76 in Cooper, 2000: 381). Therefore, while the traditional elements of hegemonic masculinity include ‘toughness, strength, and the use of force’ (Maiolino, 2015: 15), hegemonic masculinity can vary from physical prowess, such as ‘looks and athletic ability’ (Cooper, 2000: 382), to the use of vernacular speech (Kiesling, 1998), to technological expertise^6^ (Cockburn, 1988; Hacker, 1990; Turkle, 1984, 1988; all cited in Cooper, 2000). Despite these contextual variations, hegemonic masculinity always relates to status and power.

What distinguishes journalistic critics from publishers or other CIs as organizing intellectuals is that they have considerably less autonomy in the selection of cultural products. While they are important gatekeepers in reception, they deal with works that have already been chosen for publication upstream by publishers, editors, and chosen for them to review by newspaper editors (Chong, 2011: 66). This means that unlike the publishers that Childress and Nault (2019) studied, critics cannot simply ‘take a pass’ and therefore must use critical appraisal to deal with cultural products that are not a good ‘fit’. If, as DeVault contends, reviews select, translate and arrange elements of cultural products to make arguments about their social world, then what do the reviewers select from the books to reflect on and interpret gender relations in their accounts of *L’Euguélionne* and *The Handmaid’s Tale (HMT)?* In the findings section later in this article, I identify a derogatory method of reading as the discursive apparatus of the boys’ club of journalistic criticism which grants literary membership to some women based on man-centred evaluations of feminism and gendered stereotypes about artistic mastery.

## About the Books

Published in 1976, *L’Euguélionne* marks the beginning of a short, but intense period of feminist literary production in Québec between 1976 and 1980, termed the ‘Feminist Period’ (Boisclair, 1998: 208–211). While works with feminist content had been published before this date, they were not explicitly feminist, thus making *L’Euguélionne* Québec’s first unequivocally feminist novel. While the publication of *L’Euguélionne* occurred at the heart of cultural and political changes, both for women and Quebecers, *The Handmaid’s Tale* arrived on the shelves in 1985, after the heydays of feminist mobilizations in the 1970s. Nonetheless, in Canada, the 1980s, were an important period for abortion rights in the country, culminating with the legalization of abortion in 1988. Atwood’s novel tackles issues of reproductive justice and has been presented as ‘a feminist text that debates issues of reproductive technology and women’s roles’ (Wisker, 2010: 121). Both novels have also resonated with contemporary audiences through new cultural productions. In 2016, *L’Euguélionne* became the name of a feminist bookstore in Montréal which reclaimed the book as a feminist icon. Similarly, in 2017, the first season of the TV adaptation of *HMT* was released on the streaming service *Hulu* which granted the novel a second life and a new audience.

## Case Selection and Methods

Journalistic criticism, part of the *frontline* of cultural reception (Griswold and Wohl, 2015), offers an ideal venue to analyse readings of political cultural objects. Frontline criticism in newspapers is different in content and format from essayistic and academic criticism (Chong, 2011: 65). Journalistic reviews are shorter and contemporary to the production, and have a more ‘popular’ focus than essayistic or academic reviews (Chong, 2011: 65). Given media’s role as a ‘mechanism of transmission’ (Mansbridge and Flaster, 2007),^7^ journalistic review, akin to expert political or financial analyses found in newspapers, shapes the way cultural events come to be interpreted by a generalist audience (Janssen et al., 2008).

The case selection and analysis for this article is two-fold. First, when coding inductively the critical appraisal of *L’Euguélionne* in the two main Québec newspapers, I identified an overall positive reception along with recurrent challenges to the author’s creative credibility. I then chose a second comparative case to validate identified patterns in the reception of *L’Euguélionne*. The choice of *The Handmaid’s Tale* is fitting because both novels are feminist speculative fiction that share similar trajectories to iconicity. *HMT* and *L’Euguélionne* achieved recognition over time, received nominations for or won significant awards, and they were both given a second life by contemporary cultural makers. Though *HMT* reached broader critical and public acclaim than *L’Euguélionne*, partly because *L’Euguélionne* was published in a minority French context, both are best-selling novels that capture elements of second-wave feminism in their own linguistic contexts. For this second case study, the coding was done deductively, searching for different forms of creative challenges which I later conceptualized as derogation. Informed by DeVault’s concept of method of reading, I analysed the data by asking the following questions: Which aspects of the books’ feminist content do critics select and how do they engage with it? What kind of vocabulary do critics use to assess the books? What do they draw on to justify these assessments to their readers? These questions are intended to draw out the selection, translation and arrangement that go into reviewers’ accounts of the books.

Despite similarities between the two cases that make for a fruitful comparison, the two novels were published 10 years apart in different linguistic contexts, and written by authors at different stages in their careers – a debut novel for Bersianik and a sixth novel for Atwood. These differences preclude conclusions about the weight of a pre-established reputation in the reception of feminist work as well as an in-depth understanding of context effects. With no pre-established reputation, Bersianik published her novel at the height of the 1970s’ feminist mobilizations in Québec (Dumont, 2008), likely granting her more sympathetic media attention. As for Atwood, she published *HMT* at the beginning of a 10-year conservative presence in federal politics, and decreasing funding for feminist organizations in Canada, but did so with a pre-established reputation in Canadian literature thus giving her more legitimacy than Bersianik as an author, but perhaps less as a feminist. The analysis builds on these tensions between positive and negative media attention, and between a growing representation of women and an increasingly hostile context for feminist mobilization (Mendes, 2011; Rodgers and Knight, 2011). Moving away from a dichotomy between good and bad reviews based on context or reputation, which researchers have noted to be less important than merely being reviewed (Chong, 2020), I use the two cases to identify persistent patterns in the ways critics relay feminist fiction by white Canadian female authors to mainstream audiences in a context of push and pull between second wave feminist mobilization and its backlash.

The data analysed for this project primarily come from five major newspapers in Canada,^8^ in both English and French: *Le Devoir, La Presse, The Gazette, The Globe and Mail* and *The Toronto Star.*Table 1 summarizes the data sources. Amongst Québec newspapers, two are in French and one in English. *Le Devoir* is a nationalist and left-leaning newspaper known for its in-depth political analysis and critics of high-brow artistic production, *La Presse* is a federalist-leaning newspaper with a more generalist editorial orientation and *The Gazette* is Québec’s most important English newspaper. As for English publications outside Québec, *The Globe and Mail* adopts a centre-right political stance whereas the *Toronto Star* is a centrist publication (Winter, 2011). I also chose to include one article from a US newspaper and one from a Québec literary magazine in the sample because they were referenced in Canadian journalistic criticism, thus making their impact similar to other journalistic reviews.

**Table 1.** Summary of data sources and ample size (N = 82).

Location	Data source	Search word	Number of articles	Period covered by data
French, Québec-based Newspapers	*Le Devoir*	*L’Euguélionne*	27	1976–2016
		*La Servante écarlate*	8	1985–2017
	*La Presse*	*L’Euguélionne*	13	1976–2016
		*La Servante écarlate*	6	1985–2017
English, Québec-based Newspaper	*The Gazette*	*The Euguelion*	1	1976–2016
	*The Gazette*	*The Handmaid’s Tale*	15	1985–2017
English, Ontario-based Newspaper	*Toronto Star*	*The Euguelion*	0	1976–2016
	*Toronto Star*	*The Handmaid’s Tale*	3	1985–2017
	*The Globe and Mail*	*The Euguelion*	0	1976–2016
	*The Globe and Mail*	*The Handmaid’s Tale*	7	1985–2017
US Newspaper	*The New York Times*	*The Handmaid’s Tale*	1	1985–2017
Québec Magazine	*Liberté*	*L’Euguélionne*	1	1976–2016

The sample includes journalistic criticism and excludes articles that only mentioned but do not discuss the books, such as ads, best seller lists, announcements of events and award nominations. The data consist of 82 articles that span from the publication date of each novel (1976 and 1985 respectively) until the date at which they were taken up by current cultural makers who gave them a second life: a feminist bookstore for *L’Euguélionne* (2016) and a TV show for *The Handmaid’s Tale* (2017). The length of the reviews varies between as much as half a newspaper page for the longest reviews, often during the immediate context of publication, to as short as a few lines. The articles sometimes include reviews for more than one book, especially in French Québec articles. I have included these parallel reviews in my analysis as the comparisons critics make between them and *L’Euguélionne* are informative of their critical appraisal of feminist literature. Finally, for *HMT*, I have excluded all items about *HMT* the movie (1990) (i.e. showings, critiques and commentary on actors’ performances) as well as items about the bookstore *L’Euguélionne*.

The sample of journalistic critical appraisal I have meticulously constructed also tells the story of a specific era of reception for feminist novels: a period of increased feminist publications along with a relative stability in terms of how and by whom reviews are conducted.^9^ In both novels’ context of publication, namely the late 1970s and the mid-1980s, reviews are often published in the Saturday edition of mainstream newspapers, and, in their respective linguistic contexts, the two novels were reviewed mostly by male reviewers. In the case of *L’Euguélionne*, there are five articles written by women in both French Québec newspapers, the earliest of which appeared in 1979, and the pieces consist of portraits of Bersianik rather than reviews of her work. There is only one article written by a woman about *L’Euguélionne* in the *Globe and Mail* and two in the *Gazette*, all of which are about Howard Scott and his translation of the novel. The pieces written by women, about Bersianik or her translator Howard Scott, typically do not display all three components of the derogatory method of reading, but maintain the framing of Bersianik as a ‘good feminist’ (i.e. not anti-men) and often focus on the achievements of Scott. With regards to *The Handmaid’s Tale*, the sample includes one commentary piece written by a woman who references the novel to support her feminist argument, and one review that consists of a counter example of the derogatory method of reading, which I discuss in the Findings section.

The sample, as summarized in Table 1, also reveals significant attention to the translation of Atwood’s novel in French Québec media. This coverage of *La Servante Écarlate* relies on feminist cultural critics’ own selection and interest in feminist cultural productions outside of Québec, typically published a few years after the original publication of *HMT*. Given their specific interest in feminist productions, it is not surprising that their reviews cast a different light on Atwood, not showcasing derogation but rather analysing her book in dialogue with other feminist publications or in relation to her previous work. Another pattern specific to the reception of Atwood’s work in French Québec media is a discussion of her work employing the ‘two solitudes’ frame to assess anglophone Canada’s stance towards Québec literature. These articles display a generally sympathetic stance towards Atwood, likely due to her French linguistic capacities and interest in Québec politics; in the post-referendum context,^10^ being an Anglo is a more defining trait than being a feminist. In contrast, the media attention received by *L’Euguélionne* in anglophone newspapers focuses on the novel’s translator, Howard Scott. He is praised for winning the Governor General’s award in 1997 for his translation of a book famously difficult to translate due to its in-depth engagement with the gendered rules of French language.

The data presented in the following section therefore should not be understood as representative of the entirety of journalistic pieces, but rather as capturing a recurrent discursive pattern in journalistic critics that is more prominent in the early reception but present throughout the 40 years of reception. The examples provided in the following analysis consist of illustrative examples of the three components of the derogatory method of reading, an *interpretive activity* (DeVault, 1990) which shapes cultural repertoires available in discussions about women’s cultural productions.

## Findings

In this section, I present the findings of the comparative analysis of the reception of two Canadian iconic feminist novels, *L’Euguélionne* by Louky Bersianik (1976) and *The Handmaid’s Tale* by Margaret Atwood (1985), by sharing excerpts that exemplify broader patterns in the sample. I find that critics employ a derogatory method of reading – a method of reading that belittles women’s contribution, which shapes their reception and the terms of their consecration. I identify three discursive components of the method of reading: (i) a reductive reading of feminist politics, (ii) a man-centred assessment of feminism’s worthiness and (iii) a questioning of women’s creative credibility. While presented separately, these three discursive components of the derogatory method of reading work together at reproducing masculine authority as a yardstick for which feminisms are worthy of literary attention.

### On Feminism and Imagination: A Reductive Reading of Feminist Politics

Across the different contexts, historical and geographical, critics’ method of reading of both *L’Euguélionne* and *The Handmaid’s Tale* is one that defines feminist politics as unidimensional and therefore sees any feminist writing as unimaginative – merely amplifying the ‘party line’. This reading is reductive as it leaves unaccounted the literary exploration of feminist politics done by both authors. At times, such a method of reading, for *L’Euguélionne*, comes with a positive assessment, but the underlying assumption nonetheless testifies to a simplistic understanding of feminism and its relationship to literature.

For *L’Euguélionne*, the criticism about its lack of imagination is made both impli-citly and overtly by early critics who see the novel as capturing the era’s zeitgeist. *L’Euguélionne* is either enthusiastically lauded or heavily criticized for capturing the essence of Québec’s feminist mobilizations of the 1970s. The novel indeed receives two strikingly positive reviews shortly after its publication in both *La Presse* and *Le Devoir* which praise its literary quality and its contribution to Québec literature. Both critics highlight that this quality is due to Bersianik’s capacity for capturing the epoch’s zeitgeist. For Réginald Martel, the novel captures the ‘here and now’ (20 March 1976 in *La Presse*). For Jean Basile, it encapsulates 1970s feminism: ‘we can talk here of a sum: a sum of the status of women here, today’ (6 March 1976 in *Le Devoir*). The novel is thus critically acclaimed for its capacity to encapsulate the era’s feminist mobilizations.

In contrast, while the publication of a feminist novel is, in Ricard’s words, ‘monumental’ (1976: 94), according to him, the literary value of the novel should not be based on its relation to the movement.

In Québec, feminism is becoming such a ‘paradigm’, which is not a value judgement, but a simple observation, and which perhaps explains, at least partially, the critique of a work such as *L’Euguélionne*, which was celebrated like a gift from heaven, unexpected and miraculous, when in reality its impact comes precisely from the fact that it is a book literally without surprise, the exact opposite of a *nouveauté*, an expected text, especially as it formulates and summarizes perfectly what we could call ‘the era’s zeitgeist’. (Ricard, 1976: 93)

The novel’s capacity to summarize the era’s zeitgeist explains for Ricard the popular and media success, but also the book’s literary failings. For Ricard, the novel was ‘without surprises’ as it merely captured the decade’s political essence.

Being too close to the politics of everyday life has remained an obstacle to *L’Euguélionne*’s literary status even after the immediate context of publication. Ten years later, Gérard Bessette, a writer and education specialist interviewed about the *Prix Athanase-David*^11^ he just received, offers *L’Euguélionne* as a counter example of what a good novel is. To him, a good novel must be imaginative: it should ‘transport me in another reality than the one I live in’. According to him, this is not the case for *L’Euguélionne* and for women’s fiction more generally: ‘Several women authors are so obsessed with this question of women’s status that in their books, there is no distancing. However, this is necessary, I believe, to write a good novel’ (Royer, 29 November 1980 in *Le Devoir*). Unsolicited, Bessette takes his interview as an opportunity to degrade the most iconic feminist book of the last decade. Like his colleagues, he finds that Bersianik and other ‘women authors’ are not sufficiently imaginative, lacking the necessary distance from their gendered reality, to write a ‘good novel’ worthy of consecration.

Like *L’Euguélionne, HMT* is read as the summary of a political ideology, and thus as unimaginative. However, instead of lauding her for capturing the politics of her era like some critics did for Bersianik, albeit in a reductive way, this reviewer blames Atwood for it:^12^ ‘As she has said elsewhere, there is nothing here that has not been anticipated in the United States of America that we already know. Perhaps that is the trouble: the projections are too neatly penciled in.’ (Mary McCarthy, 9 February 1986 in *New York Times)*. McCarthy later concludes that the novel ‘lacks imagination’, a criticism that gets taken up by Richard Marin, a Canadian critic who contends McCarthy got to ‘the heart of the matter’ (31 January 1987 in *The Gazette*). This intertextuality of criticism demonstrates the kind of social orchestration at play in critical appraisal (Chong, 2011). That critics agreed on Atwood’s lack of imagination is not trivial as it is used by male critic Clark Blaise in *The Gazette* to withhold the kind of literary consecration granted to Orwell, a male author of a similar genre:

What keeps *The Handmaid’s Tale* from the company of Orwell (or keeps me from appreciating it as fully as I might), is the imbalance of imagination, the familiarity of the villains in their right-wing, missionary-positioned religiosity, with all their predictable fetishistic repressions. (Clark Blaise, 5 October 1985, in *The Gazette)*

Vocabulary used by Blaise here such as ‘familiar’ and ‘predictable’ recalls that of Montréal poet Solway who, much like Bessette did for *L’Euguélionne*, went out of his way to qualify Atwood as ‘dull, predictable and derivative’ (Thomas Schnurmacher interview with Montréal poet Solway in *The Gazette*, 2 October 1989). Such derogatory terminology is gendered, as women, and feminists in particular, often encounter those disparaging characteristics (Ahmed, 2012; Kein, 2015).

Finally, Atwood receives contradictory reviews about her tackling of feminist politics: she has too much ambition and is also cutting herself short. These contradictory reviews are linked by critics’ reductive reading of politics in literature. For William French, the problem is not in Atwood’s treatment of politics, for instance the way she exposes power dynamics and feminist ideas through her characters and their storyline, but rather that she did so at all. As French explains in his critique, ‘[t]he attempt doesn’t entirely succeed, but that’s due more to her excessive ambition than to any flaws in execution’ (William French in the *Globe and Mail* in 1985). This criticism goes hand in hand with the reductive reading of politics because if Atwood’s book is the summary of a political ideology, then embarking on such an exegesis signals she is ‘over-reaching herself’ (William French in the *Globe and Mail* in 1985). In contrast, looking for a single villain and an action plan, McCarthy blames Atwood for being insufficiently prescriptive. As she summarizes the plot in her review, she says that ‘the book just does not tell me what there is in our present mores that I ought to watch out for unless I want the United States of America to become a slave state something like the Republic of Gilead whose outlines are here sketched out’ (Mary McCarthy, 9 February 1986 in *New York Times*). In both reviews, the exploration of feminist politics that Atwood embarks on through the dystopian future she creates is left unaccounted for and instead used as evidence of her desperate ambition or her failings at imagining a remedy.

Overall, *L’Euguélionne* and *HMT* were met with mostly negative reviews due to the critics’ reductive reading of feminist politics. However, what is at stake here is not so much the positive or negative character of the foregoing reviews – it is, after all, a reviewer’s job to be critical – but the reductive way they treat the feminism and its relationship to literature. The novels are read as summarizing feminist politics, which for some critics leads to negative reviews blaming the lack of imagination and originality, while for others, it is cause for praise which leads to laudatory reviews of the book’s capacity to capture the era’s zeitgeist. This reveals that despite reading the books in a context that provides them with intellectual resources to capture the importance of feminist literature,^13^ critics are not well equipped to assess political art. By reading literature as a simple capsule of political ideology, the books become a convenient receptacle of all that feminism is which, as I will show next, opens the door for critics’ assessment not only of the books, but of the movement.

### Lecturing, Challenging and Silencing: Man-centred Assessments of Feminism

The reductive reading of feminist politics identified in the previous subsection makes it seem as though critics do not engage with the feminist ideas put forward by the books. However, they do engage with them, but only to assess the value of the movement based on their own experience. In fact, this assessment takes over that of the book itself. The critics’ own feeling of exclusion from feminism, be it in the book or the movement more broadly, ensures authors a negative review. What is more, portraying the ‘wrong’ kind of feminism can cost the authors not only a good evaluation, but also the expected attention to their literary proposition. For some critics, feeling lectured or excluded by feminist politics makes the books go from unimaginative to unworthy of literary attention.

Reviewers who felt lectured or targeted by *L’Euguélionne*’s feminism criticized the author’s writing style. A problematic element about *L’Euguélionne* for Ricard, writing in 1976 soon after the publication of the novel, is his feeling of being lectured during his reading. He explains his dislike of the writing style based on: ‘the unpleasant impression of being taken for a school student’ (Ricard, 1976: 93–94). This is related to the book itself, but also to feminism more broadly as he pre-emptively targets the movement for censoring his criticism: feminism has become such a ‘paradigm’ in Québec that he is ‘forced into silence’.

The feeling of being lectured is constant through the years as *Le Devoir* critic Cornellier, writing in 2007, reviews similarly two newly published feminist works: a translation of Andrea Dworkin’s essays and a collection of essays by Bersianik. Like Ricard in 1976 when reviewing *L’Euguélionne*, Cornellier also feels he is being lectured by Dworkin’s *Pouvoir et violence sexiste* and connects this reading to feminism beyond the book he is reviewing. He does so by explaining how hurtful people’s perception of him felt after the Polytechnique^14^ mass shooting. He equates his feeling of being lectured by Dworkin’s collection of essays to the way he felt the day after the Polytechnique shooting when he was put in the ‘bad camp’ by radical feminists at UQÀM (Université du Québec à Montréal).^15^ This feeling of being wrongly accused takes up all the space in his review as he talks about the content of the book only to bring it back to his exclusion from her feminism. He concludes the segment dedicated to the review of Dworkin’s book with a statement about the trajectory feminism should take: ‘By her radicalism and her exaggerations, Andrea Dworkin excludes me from a movement that is also mine. That is not where the future of feminism lies.’ Therefore his ‘refusal . . . to be assimilated to the bad guys’ blinds everything else, making the literary review a secondary concern to the assessment of feminism.

In contrast to Dworkin, Bersianik’s collection of essays, published in 2007 and reviewed in the second half of Cornellier’s article, receives a euphemized criticism of her feminism. Bersianik is given some literary attention because, unlike Dworkin, she portrays a more acceptable feminism. While Cornellier takes issue with Bersianik’s view on masculinity, he acknowledges that, in line with her first novel *L’Euguélionne*, she offers ‘an interesting mythological and feminist re-reading of human history’. Similarly, other critics saw Bersianik’s first book as worthy of praise because it is not anti-male: ‘*L’Euguélionne*, indeed . . . does not put aside men, though she often judges them with severity’ (Basile, 6 March 1976 in *Le Devoir*). He goes on to say that the book is the ‘right’ kind of feminist literature, that is, ‘not a literature that rejects men, a literature where men do not have their place. Some other feminist authors, from here and abroad, often see ‘men’ like an enemy and treat them as such’ (Basile, 6 March 1976 in *Le Devoir*). The derogatory method of reading thus works insidiously here. Writing 30 years apart, both Cornellier and Basile give praise to Bersianik, but they do so by establishing a prerequisite that feminists must comply with before they can access such literary praise. Being interpreted as a non-threatening feminist by male critics thus makes Bersianik’s feminism palatable, while other versions, like Dworkin’s, is inacceptable.

Not being read as anti-male feminist, a key component in critics’ assessments of the book’s quality, varied across reviewers and benefited Bersianik more than Atwood. Critics of *HMT* took the book’s feminism as a personal assault: ‘I feel a disservice has been done me. We’ve been patronized!’, Blaise writes in *The Gazette* (5 October 1985). Much like for *L’Euguélionne*, the feeling of being lectured is a major obstacle for the critics’ enjoyment of *HMT*, whose politics make it pedantic as ‘[t]he bones of contention [poke] through the narrative skin’^16^ (5 October 1985). For French, writing soon after the publication of Atwood’s novel, the book is a sermon with an unclear message:

The point of the sermon? Atwood seems to be saying that the present, with all its excess, options, decadence, abuses and moral bankruptcy, is better than a future without freedom. She won’t get much argument there. (William French, 5 October 1985 in *Globe and Mail*)

While French uses religious terms to frame *HMT* as pedantic, other critics frame *HMT* in terms of litigation: Atwood is ‘listing “exhibits” in her case against patriarchy’ (Marin, 31 January 1987 in *The Gazette*) and her book consists of a ‘permanent complaint’ (Symons’ words are reported in a piece by Mark Abley in *The Gazette* on 23 June 1990).

The foregoing findings confirm what Childress and Nault (2019) have found regarding the reliance on personal experience in evaluation in art worlds. Male critics rely on their own masculine experience of feminism to assess the worthiness of the books. What the findings also reveal is the consequence of a lack of fit during the 1980s and 1990s in Canada between the critics’ interpretive lens, and the literary proposition of feminist authors. This gap transforms reviews into accounts of critics’ hurt feelings – how they are being schooled, preached to or challenged – and become assessments not only of the books, but of the movement, specifically the way it treats men. Reviewers read feminism, in both the book and society, as an affront to their authority and react to it by assessing the worthiness of the cause. Their feeling of being challenged and excluded determines whether the authors are the ‘right’ kind of feminist, which in turn determines whether they are worthy of literary attention.

### Questioning Women’s Creative Credibility

In this final subsection, I further show how gender plays into the reviewers’ disparagement of the books as I highlight how reviewers draw from the gendered character of expertise as masculine (Cooper, 2000: 38) to challenge women’s creative credibility. This translates into a disbelief in critics’ reviews that cast doubt over the literary value of the feminist books. In their reviews, critics question women’s creative choices about their personae as authors, criticize their proficiency in the genre and challenge the speculative futures they create.

Critics’ questioning of feminist authors’ choices is, for *L’Euguélionne*, centred on the use of her pen name – Louky Bersianik instead of Lucille Durand:

Her real name is Lucille Durand. Anecdotally, let us mention as well that she did not come out of nowhere, as the wife of a CBC director and sister of a well-known actor, she has certainly been mingling with Montréal’s intellectual circles. Moreover, she is not a little girl, stuck in her inner turmoil, but a grown woman. Why is she hiding behind this exotic penname? I do not know. To better defend the cause of the book, maybe. (Jean Basile, 6 March 1976 in *Le Devoir*)

Basile presents Bersianik in relation to famous men in her life, who in turn connect her to Montréal’s intellectual circles. It is suspicious of her to cover up those connections with a pen name, and it would only make sense to use one if she were a troubled young woman. The rhetoric of derogation works subtly, as the offence is not directly aimed at her literary capacity or the novel’s quality. Nonetheless, the suspicion consists of an additional hurdle towards consecration: Bersianik must first convince the reviewer that she can be trusted as an author, and then convince him she is one of talent.

The book’s genre is also at stake for critics who remain unclear about where it ‘truly’ belongs. Basile says it ‘could’ be science-fiction, though for him, it resembles more the ‘bible’s preaching style’. The biblical reference continues, but less favourably with Ricard who qualifies the novel’s style as ‘philosophico-theologico-judicial-scientifico-elementary speeches and sermons on the mountain’ (Ricard, 1976: 93–94). While Ricard’s and Basile’s doubts were cast in reviews at the time of publication, we continue to find such doubt in reviews almost 40 years after its publication. For instance, Cornellier holds that Bersianik herself ‘cultivates a certain obscurity’ because of the references she makes in *L’Euguélionne* to ancient Greece and its mythology, a topic about which he questions her expertise. This is expressed by the conditional tense in French which translates to a supposition: ‘cultivating at times a certain obscurity, maybe due to the multiple unexplained analogies to ancient Greece *supposedly* well known by Bersianik’ (emphasis added). Cornellier casts doubt over Bersianik’s expertise and blames her for his own lack of knowledge about the book’s references. This leads him to conclude with a lukewarm assessment of Bersianik’s inclusion in a Québec literary anthology, neither agreeing nor opposing it by using a double negative: ‘I am not a fan, but I do not disagree [with her consecration]’. The suspicion towards Bersianik’s expertise is key in the procedures of derogation. As was the case for the reading of politics as unimaginative, whether the critics frame their doubt as positive or negative is not at stake. Rather, it is the questioning itself – of her penname, the genre and her expertise – and their attempts at solving that uncertainty in their reviews that contribute to the belittlement of her feminist writing.

For *HMT*, the doubt is about the fictional world Atwood builds which sparks a fact-checking impulse amongst critics. For instance, in her *New York Times* review (9 February 1986), Mary McCarthy shares her disbelief about the realization of Atwood’s dystopian future:

I can’t see the intolerance of the far right . . . as leading to a super-biblical puritanism by which procreation will be insisted on and reading of any kind banned. Nor, on the other hand do I fear our ‘excesses’ of tolerance as pointing in the same direction.

This doubt is surprising given the book’s genre; in speculative fiction, and particularly in works that lay out a dystopian future, social problems are often amplified, ‘taken several steps further to their logical and dark conclusion’ (Wisker, 2010: 24) to illuminate problematic aspects in the current state of affairs.

In most reviews, the distrust is not cast as widely as in McCarthy’s review, but rather pertains to the portrayal of men and masculinity. One critic from *The Gazette* sees the female narrator as a ‘credible contemporary woman’ with ‘insight and eloquence’ but does not agree with the portrayal of male characters: ‘My reservation is with the picture of that masculinity’ (Blaise, 5 October 1985 in *The Gazette*). He explains:

Commander [Fred] and [Serena Joy] are oafs, and the imagined apparatus of oppression is hardly worthy of the great advances in criminality and behavior-modification pioneered by the modern state (or imagined, by Orwell among others). In other words, bad as Fred and the others are, I don’t believe they are bad, frightening or subtle enough. (Blaise, 5 October 1985 in *The Gazette*)

Dystopia as a genre, be it for Orwell or Atwood, is used to dramatize themes of control, domination and oppression (Wisker, 2010: 129) which renders absurd questions about the accuracy of Gilead and its protagonists. Nonetheless, the critic’s questioning of her capacity to ‘realistically’ imagine villains and an apparatus of oppression casts doubt over her worthiness as an author, unlike a consecrated male author of the same genre.

Another critic in *The Gazette* in turn questions what he views as stereotypical representations of men and women, which leads Atwood to miss the ‘slippery ambiguities’ of the erotic: ‘By insisting on a programmatic male–female dualism, Atwood cramps the imagination. Language is not simply the rationalistic tool of man’s invention, nor does man always occupy the surface of myth, woman the underground’. This translates into a negative critique of Atwood’s style: ‘Ironically her writing is, by her own standards, unquestionably “masculine” – didactic, calculating, anemic’ (Marin, 31 January 1987 in *The Gazette*). The disbelief of her portrayal of masculinity does not happen in a vacuum, disconnected from the other discursive elements of the derogatory method of reading. The incredulity is expressed in parallel to the assessment of her feminism – the ‘wrong kind’ that stereotypes men and restricts the imagination.

Two contrasting examples found in the data demonstrate what granting the author creative credibility allows in a literary review. Reporting on a recently published anthology of feminist science fiction reviewed in *The Globe and Mail*, the reviewer writes:

The manipulation of genres that results – whether it’s the ‘speculative fiction’ of Atwood’s *The Handmaid’s Tale*, or the confessional fiction of Barfoot’s *Dancing in The Dark* or the near-Harlequin *Romance of Engel’s Bear* – suggests a uniquely feminine awareness of how the old forms no longer serve to describe the realities of women’s lives. (28 November 1987 in *Globe and Mail*)

The critic follows the author of the anthology as they grant Atwood creative credibility and wilfully suspend their disbelief about the veracity of the fictional world that’s being created. This allows the critic to consider how the form and genre employed by Atwood contribute to feminist political thought. Another similar example, not scholarly this time, is *Toronto Star*’s Adachi who connects *HMT* to Atwood’s previous works to explore her trajectory as a writer and set up a comparison: ‘But it’s really quite different from anything she has written previously. Here she takes us into another time and another country, one beyond Canada and into an imagined state.’ The suspension of disbelief in both these cases allows the reviewers to situate Atwood within an established sub-genre of feminist writing and to track her trajectory as an author, rather than employ the rhetoric of derogation by hashing out the specifics of Atwood’s dystopian future and feminism.

In sum, critics employ a method of reading both books that understands them as derivative, summarizing rather than creating feminist politics. This leads to both positive and negative, but always reductive, readings. It is either treated as an exploit, such as journalists’ praises of Bersianik’s capacity to capture the 1970s zeitgeist in Québec, or as too ambitious and unimaginative for journalists reviewing *The Handmaid’s Tale*. This reductive reading of politics leaves unaccounted the complexity of the dystopian realities and of feminist politics in both books. This in turn makes the reviewers focus on feminism itself and their own assessment of it, rather than the authors’ contribution to the movement. Feeling excluded and at times challenged by the books’ feminism sparks a fact-checking impulse amongst critics. The questioning of the authors’ creative credibility is particularly salient when it comes to their portrayal of masculinity, showing how, like other uses of dystopia and irony, it is often ‘misread by those who it ironized’ (Wisker, 2010: 24).

## Discussion and Conclusion

As a field where physical prowess and economic power are not the main currency, how is hegemonic masculinity articulated in art worlds? The analysis of the reception of *L’Euguélionne* and *The Handmaid’s Tale*, two Canadian novels of feminist speculative fiction, demonstrates that critics evaluate feminism based on masculine experience and relay gendered stereotypes about artistic mastery through a derogatory method of reading that belittles these authors’ contribution. The method of reading is founded on three components: (i) a reductive reading of feminist literature, summarizing rather than theorizing feminist issues; (ii) an assessment of feminism based on the critics’ own experience of the movement; and (iii) a challenge to women’s creative credibility through fact-checking impulses which cast doubt over the quality of their work. These findings illuminate how women’s work is not openly discarded through vindictive or negative evaluations based on gender (nor fought against through laudatory ones), but rather belittled by implicitly gendered claims even as they are granted access to consecration.

While presented separately, the three discursive components of the derogatory method of reading worked together at minimizing the authors’ aesthetic and creative contributions. The findings indeed revealed how criticism of Bersianik’s lack of imagination was intertwined with critics’ feelings of being lectured, which critics dislike and blame the author for. In turn, Atwood’s ‘predictability’ is interlocked with the disbelief of the dystopian future in *HMT*, translating into a fact-checking impulse about the imagined world she creates. In sum, journalistic critics fault both authors for their unimaginative summary of feminism, but also deny the plausibility of the speculative realities they are presenting. Speculative fiction makes absurd critics’ incredulousness, which demonstrates how genre has gendering power. Indeed, genres ‘erect and control the boundary between what is permissible or prohibited for writers’ (Alacovska, 2017: 382). In the case of Atwood and Bersianik, they crossed the gendered genre boundary by using speculative fiction to draw a critical portrait of masculinity. Reviewers punished such ‘creative inappropriateness’ (Alacovska, 2017: 382) not openly, but rather indirectly by withholding their suspension of disbelief.

The concept of method of reading propels the study of gender and reception beyond individual bias by showing patterned ways in which critics use their interpretations of books to make arguments about their gender and feminism. In reviewing the literature on CIs, I have identified three features distinct to the work of critics that give their gendered experience significant weight: the epistemological uncertainty, the reliance on personal experience and the importance of fit. This suggests a dynamic akin to Delvaux’s concept of boys’ club, where notions of membership, talent and legitimacy are defined along gendered lines. As for the context, both books were published after major feminist mobilization and increases in feminist publications which forced an encounter with fiction that directly challenged their authority, within and beyond the literary field. Not finding a good fit between their predominantly masculine experience and that presented to them in feminist speculative fiction, critics employ a discursive arsenal that belittles feminist authors’ contributions to make sense of this cultural *mis*match. Finally, while context is known to influence the reception of a book (Corse and Westervelt, 2002; DeVault, 1990), the pattern of derogation is constant through the time and linguistic contexts. This consists of ‘coercive gendering’ (Alacovska, 2017: 392), which grants literary attention and merit only when authors correspond to the appropriate expression of feminism. The derogatory method of reading as a mechanism of coercive gendering helps to explain the findings of other scholars who find that *some* women, such as anglophone women in the Booker prize award (Childress et al., 2017), get consecrated. Feminism, when white and written by writers who belong to colonial categories, is more palatable and has only the hurdle of men’s sensitivities to face on the path to consecration.

I have also argued that the orchestration of literary criticism along masculine standards of artistic mastery casts CIs as the organizing intellectuals of hegemonic masculinity in art worlds and the derogatory method of reading they employ as part of the discursive apparatus. In Connell’s original use of Gramsci’s concept of hegemony, they highlight the role of organizing intellectuals, that is, actors who ‘regulate and manage gender regimes; articulate experiences, fantasies and perspectives, reflect on and interpret gender relations’ (Connell, 1983 in Donaldson, 1993: 646). Put simply, they are responsible for the ‘weav[ing] of the fabric of hegemony’ (Connell, 1983 in Donaldson, 1993: 646). Reference to the literature on hegemony in the analysis of critical appraisal thus draws out the organized character of masculine domination, and pulls out attention to the practices and discursive apparatus dominant actors must deploy to legitimize and reproduce their power and status. While critics’ domination is not defined by traditional markers of hegemonic masculinity like economic wealth or physical strength (Maiolino, 2015: 15), they can nonetheless be usefully conceptualized as a boys’ club to highlight the role they play not only in consecrating some literary works, but also in articulating for their audiences which gender experiences are worthy and which representations of reality are valid.

How could future research make sense of the implicitly gendered, but powerful ways in which the boys’ club of literary criticism belittles second-wave Canadian feminist speculative fiction? I see my findings as having implications for two main areas: for future analysis of power relations and reception, and for a critical reflection on diversity in art worlds.

First, the analysis of the discursive style used to belittle Atwood’s and Bersianik’s contribution is an invitation for the analysis of the politics of cultural discourse in the reception of marginalized artists’ production. As discussed earlier, the derogatory method of reading is not the sole product of individual bias – the result of a few bad apples. Rather, it is sustained by organizational gendered practices that make masculine experiences of the world the yardstick for legitimacy. I posit that the more writers stray from this masculine (and white^17^) standard, based on political affiliation, gender or race, the more likely the mechanics of derogation will be used in the appraisal of their work. Reductive readings of artists’ realities, assessments of political art based on hegemonic experiences, as well as challenges to artists’ creative credibility are thus likely to be present more widely than for feminist books. These accounts re-enact stereotypes about who is capable of artistic mastery and, because of the pivotal role or CIs in reception, they relay derogatory lenses for the reading of other cultural productions. I introduce the concept of boys’ club and its derogatory method of reading as a framework for the analysis of orchestrated discursive practices in reception that shape collective repertoires available to discuss non-hegemonic artists’ work and identities.

Second, this study sheds light on the question of diversity in art worlds. Social movements and art-world actors themselves have lamented the stubbornly hegemonic character of art worlds, and contemporary debates around diversity call for an effort to increase the representation of traditionally marginalized groups. This mirrors discussions of gender quotas in politics (Clayton and Zetterberg, 2018) and affirmative action in higher education (Skrenty, 2006; Stulberg and Chen, 2013). The findings presented here reveal that we should also attend to the disparaging ways in which the stories of those who compose such diversity are read, interpreted and evaluated. This study thus compels us to take seriously the impact on people’s lives and careers that may come with the scrutiny of being the diversified façade of an otherwise unchanging institution.

## Notes

1. Curated by a black woman and a white woman, most artists were people of colour and half were women.

2. *L’Euguélionne* is the debut novel of Louky Bersianik. Because of its parody of the Bible as well as its considerable size, a hefty 400 pages, the novel has been qualified as ‘the bible of Québec’ feminism. Equally funny and radical, *L’Euguélionne* is an essay-cum-science fiction novel which constitutes ‘an encyclopaedia of patriarchal culture seen from a radical feminist point of view’ (Smart in Bersianik L, 2012 [1976]: 9).

3. *The Handmaid’s Tale* was published in 1985 by the internationally renowned Canadian author Margaret Atwood. It is a speculative fiction novel that explores the themes of women’s reproductive roles, feminism, social power and control, as well as religion.

4. See Corse and Westerwelt (2002) and DeVault (1990) for analyses of context’s influence on reception, particularly the effects of anti-racist and feminist mobilizations.

5. I am thinking in particular here oft Chong’s *Inside the Critics’ Circle* (2020), where she innovatively borrows from the analytical approach of cultural production to look at the process of reviewing, as well as Childress’s *Under the Cover* in which he follows a book through the interlocking fields of creation, production and reception (2017: see in particular pages 8–11).

6. Take for instance the case of Silicon Valley, where coding the best code earns male employees their hegemonic status (Cooper, 2000: 382).

7. Mansbridge and Flaster’s (2007) work presents a feminist version of the media transmission dynamic explored in this article as they explain how a denunciatory expression coined by enclaves of organized feminist activists got transmitted to everyday activists through print media.

8. Exact numbers for the daily circulation of each newspaper are difficult to access for the entire period of the sample, but in 2013, Québec newspapers (*Le Devoir, La Presse* and *The Gazette*) had respectively a daily circulation of 33,000 copies, 218,000 copies and 130,000 (Laxer and Korteweg, 2016). As for newspapers outside Québec, per the *2013 Daily Newspaper Circulation Report*, the average weekday and Saturday paid circulation for the *Toronto Star* are 220,231 and 342,306, and for *The Globe and Mail* 259,659 and 317,971.

9. This is a pattern also identified by Boisclair (2019) in the Québec literary field.

10. Québec held two referendums on independence: one in 1980 and one in 1995. The first referendum resulted in a ‘No’ majority of 59.56%.

11. The *Prix Athanase-David* is a literary award presented annually to a Québec writer to honour the body of their work. It is one of the *Prix du Québec* awards that the Québec government presents to honour cultural and scientific achievements.

12. This is likely due to a changed context for feminist mobilization in the mid-1980s. As Corse and Westerwelt (2002) explain, intellectual resources used in order to read a book depend on context. While the goal of this article is to identify ongoing derogation, that is, over time beyond context effects, it is nonetheless worth noting that the effervescence around the 1970s’ mobilizations in Québec, including feminist and nationalist movements, likely made *L’Euguélionne*, a feminist novel published by well-known nationalist activist Hubert Aquin, cause for celebration.

13. DeVault (1990) and Corse and Westerwelt (2002) show that mass mobilization gave critics a new interpretive lens through which to interpret and evaluate the stories and the character development in books featuring characters from marginalized communities.

14. The Polytechnique shooting refers to an anti-feminist shooting that happened in an engineering school in Montréal on 6 December 1989. The shooter, Marc Lépine, killed 14 women and injured 13 other people. This shooting is a pivotal moment in Canadian feminism and its commemoration has been central to the movement.

15. The reference to Polytechnique is likely due to the fact that one of the essays in *Pouvoir et violence sexiste* is the speech Dworkin gave in Montréal on 6 December 1990 at the one-year commemoration events of the shooting.

16. Original quotation was modified to improve flow: ‘The bones of contention are poking through the narrative skin’ (5 October 1985).

17. While race has not been a core focus of this analysis since both authors are white, the boys’ club, in Delvaux’s definition of the term, is characterized by masculinity as well as whiteness which have come to be perceived as neutral (Delvaux, 2019: 120).
